# Laparoscopic Pectopexy versus Vaginal Sacrospinous Ligament Fixation in the Treatment of Apical Prolapse

**DOI:** 10.3390/life13101951

**Published:** 2023-09-23

**Authors:** Simona Brasoveanu, Razvan Ilina, Ligia Balulescu, Marilena Pirtea, Cristina Secosan, Dorin Grigoraș, Daniela Chiriac, Răzvan Bardan, Mădălin-Marius Margan, Alexandru Alexandru, Laurențiu Pirtea

**Affiliations:** 1 Department of Obstetrics and Gynecology, Victor Babes University of Medicine and Pharmacy, 300041 Timisoara, Romania; brasoveanu_simona@yahoo.com (S.B.); ligia.balulescu@umft.ro (L.B.); marilenapirtea@yahoo.com (M.P.); cristina.secosan@gmail.com (C.S.); grigorasdorin@ymail.com (D.G.); danielachiriac98@yahoo.com (D.C.); laurentiupirtea@gmail.com (L.P.); 2 Department of Surgery, Discipline of Surgical Semiology II, Victor Babes University of Medicine and Pharmacy, 300041 Timisoara, Romania; 3 Department of Urology, Victor Babes University of Medicine and Pharmacy, 300041 Timisoara, Romania; rbardan@yahoo.com; 4 Department of Functional Sciences, Discipline of Public Health, Victor Babes University of Medicine and Pharmacy, 300041 Timisoara, Romania; margan.madalin@umft.ro; 5 General Medicine, Victor Babes University of Medicine and Pharmacy, 300041 Timisoara, Romania; alexandru.alexandru@student.umft.ro

**Keywords:** laparoscopic pectopexy, apical prolapse, sacrospinous ligament fixation

## Abstract

Objectives: To compare the follow-up results of a sacrospinous ligament fixation (SSLF) technique for laparoscopic bilateral fixation of the vagina to the iliopectineal ligament via a PVDF-mesh (laparoscopic pectopexy technique, LP) in terms of cure rate and postoperative complications rate. Material and methods: This prospective study included 160 patients diagnosed with pelvic organ prolapse stage II–IV according to the POP-Q system. Eighty-two patients (51.25%) underwent vaginal sacrospinous ligament fixation and seventy-eight patients (48.75%) underwent the laparoscopic pectopexy procedure. Results: The cure rate was high in both groups, 95.12% of the patients (78 out of 82) in the SSLF group and 93.59% of the patients (73 out of 78) in the LP group were cured post surgery, leading to an overall cure rate of 151 out of 160 patients. Pelvic pain was present in 5.00% of all patients, but was notably more frequent in the SSLF group (7, 8.54%) than in the LP group (1, 1.28%). Dyspareunia occurred in 4.37% of all patients, slightly more frequently in the SSLF group (6, 7.32%) than the LP group (1, 1.28%), but without significant difference. Conclusions: The laparoscopic pectopexy procedure has comparably positive follow-up results with the conventional sacrospinous ligament fixation procedure. Both SSLF and LP are effective in the treatment of pelvic organ prolapse, with favorable anatomical and subjective results, a high cure rate and low rates of serious postoperative complications.

## 1. Introduction

Pelvic organ prolapse (POP) is a common gynecological health issue with an estimated prevalence of up to 50% by vaginal examination, and 3–6% by reported symptoms [1]. Despite being the least common POP type, with a range of 5–15%, apical vaginal support is thought to be the keystone of pelvic organ support [2]. By 2050, it is anticipated that there will be 4.9 million cases of pelvic organ prolapse given the aging population [3].

Depending on the severity of the prolapse and the symptoms, there are many treatment options. Simple observation, vaginal pessaries, or different surgical techniques are used to treat POP including vaginal, open abdominal, laparoscopic, and robotic procedures, the use of native tissue repair or graft augmentation, and based on whether uterine preservation is desired or not [4].

The technique of sacrospinous ligament fixation (SSLF) was initially introduced by Amreich in 1950 and then modified by Richter in 1968 with the aim of addressing the fixation of the vaginal apex to the sacrospinous ligament [5]. SSLF has a well-established and extensive history as a surgical procedure for the management of vaginal vault prolapse. The procedure has demonstrated consistently high treatment success rates and has become a commonly utilized surgical approach in the field of pelvic organ prolapse [6]. The use of surgical mesh for transvaginal POP surgery was reclassified by the US Food and Drug Administration (FDA) in 2016 as a class 3 procedure (high risk). Native tissue repair in vaginal surgery and laparoscopic procedures has become more significant since this FDA reclassification [7]. Native tissue repair in vaginal surgery removes mesh-related complications such mesh erosion and infection. This is crucial for women who are at an increased risk of mesh erosion, such as those who smoke, take immunosuppressive medications, or have uncontrolled diabetes [6,8].

The importance of minimally invasive surgery for POP treatment has increased due to the laparoscopic procedure’s potential benefits. Noe introduced the laparoscopic pectopexy as a new surgical technique for apical repair in 2007. The mesh is fixed with this technique using the iliopectineal ligament on both sides. According to research by Cosson and colleagues, the sacrospinous ligament and the arcus tendineus of the pelvic fascia are statistically substantially weaker than the iliopectineal ligament [8,9]. A protective effect against de novo central and lateral cystocele has been reported as an important advantage of LP [5].

Objective: To compare the follow-up results of the sacrospinous ligament fixation (SSLF) technique for laparoscopic bilateral fixation of the vagina to the iliopectineal ligament via a PVDF-mesh (pectopexy technique) in terms of cure rate and postoperative complications rate: de novo cystocele, de novo rectocele, pelvic pain, de novo constipation, de novo SUI, dyspareunia and de novo urgency.

## 2. Materials and Methods

### 2.1. Patient Selection and Inclusion Criteria

We performed a prospective cohort evaluation including all eligible patients (160 patients) who underwent surgery for POP stage II–IV in the Department of Obstetrics and Gynecology of Timisoara University City Hospital, between January 2015 and December 2020. The patients included in our study underwent either laparoscopic pectopexy or vaginal SSLF procedure.

The study was completed after obtaining the permission from the Human Ethical Committee of the Victor Babes University of Medicine and Pharmacy, Timisoara, Romania (Nr. 32/2015) in accordance with ethical standards. All procedures performed in this study involving human participants were in accordance with the Helsinki Declaration (as revised in 2013). Written informed consent was obtained from the patients.

The following parameters were evaluated for each patient: BMI, parity, post-menopausal and postoperative complications.

The follow-up period comprised evaluation at 1, 12 and 24 months after the procedure. The follow-up visits targeted the following parameters: de novo cystocele, de novo rectocele, pelvic pain, de novo constipation, de novo SUI, dyspareunia and de novo urgency.

Inclusion criteria: patients diagnosed with POP-Q II–IV according to the pelvic organ prolapse quantification system (POP-Q) were eligible for the study and the degree of the genital prolapse was assessed via a physical examination for prolapse quantification.

Exclusion criteria: patients who refused surgical treatment, patients not suitable for the surgery, patients with uterine pathology associated with indication for hysterectomy, patients with symptomatic cystocele and patients with SUI were all excluded from the study, because the complete surgical treatment would require the association of the other techniques such as anterior colporrhaphy or transobturator tape, and the impact of the technique on the apical defect would be more difficult to assess through outcomes.

The indications for both procedures were the same. Patients, in general, complain about symptoms related to vaginal protrusion or associated symptoms of the urinary bladder.

The patient had also been examined when lying down. This preoperative assessment is important to prevent an over- or under-correction and can also sometimes give information about the quality of the tissue.

Both procedures were performed by the same surgical team. A single dose of antibiotic for prophylaxis was administrated preoperatively. The Foley catheter was removed 12–24 h after surgery in the SSLF group and 24 h after in the laparoscopic pectopexy group.

**Figure 1:**
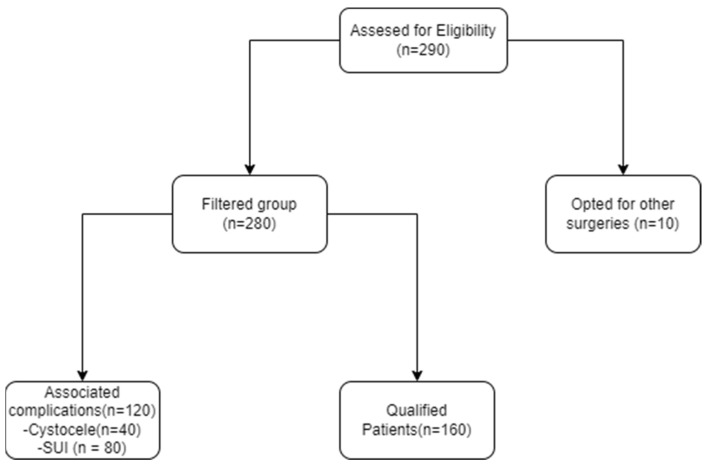
The algorithm of patient recruitment.

The algorithm of patient recruitment (Figure 1): 

Outcomes: The cure rate was assessed by vaginal examination. The success of surgical treatment was defined as the position of the cervix being above the level of the hymen.

Validated questionnaires such as the Prolapse Quality of Life (P-QOL) questionnaire were completed at baseline and follow-up visits. The Urinary Distress Inventory-6 (UDI-6) questionnaire was completed just for patients who presented de novo SUI and de novo urgency post operatively.

#### 2.1.1. Technique Description for Laparoscopic Pectopexy

The patient was set up in a specific position for the procedure: lying on the table at a 45° angle in the Trendelenburg position, with hips extended, knees flexed, and in a dorsal lithotomy posture. To facilitate the process, a 16-F catheter with a 10 mL balloon tip was carefully inserted into the bladder and connected to a continuous drainage system.

The introduction of pneumoperitoneum was achieved using a direct entry method. An endoscopic camera was then inserted through the first trocar, which was a 10 mm trocar placed in the umbilicus. With clear visibility over the lower abdomen, three additional 5 mm laparoscopic trocars were inserted in the standard positions. One 5 mm trocar was positioned 5 cm below the umbilical trocar, while two 5 mm access ports were placed 2 cm medial and above the anterior superior iliac spine on each side.

To assist with the procedure, a uterine manipulator was introduced into the vagina and held in place by an assistant, effectively exposing the anterior vaginal wall.

**Figure 2:**
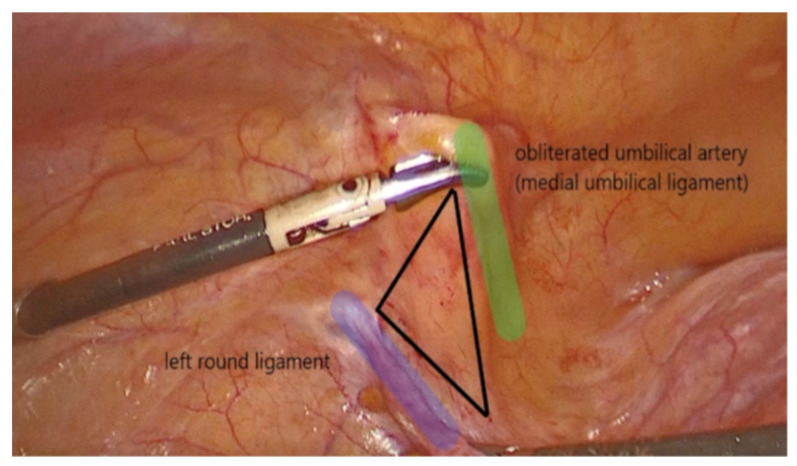
V Shape.

**Figure 3:**
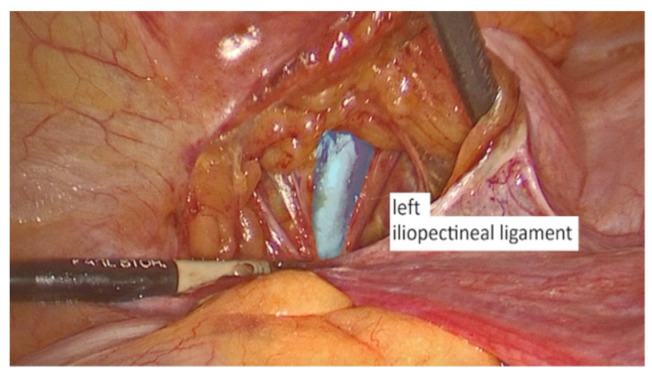
Iliopectineal ligament.

The dissection started on the left side of the pelvis. The left round ligament and the obliterated umbilical artery formed a V shape around which the peritoneum was incised (Figure 2). The left iliopectineal ligament (Figure 3), commonly known as the inguinal ligament of Cooper, was located directly under the external iliac vein and prepared during the soft tissue dissection. On the right side of the pelvis, the identical steps were performed.

The peritoneal incisions on both sides were enlarged superficially via a blunt dissection that followed an imaginary line between the physiological axis of the pectineal line and the anterior peritoneum of vesico-vaginal space after the iliopectineal ligament had been found and prepared.

**Figure 4:**
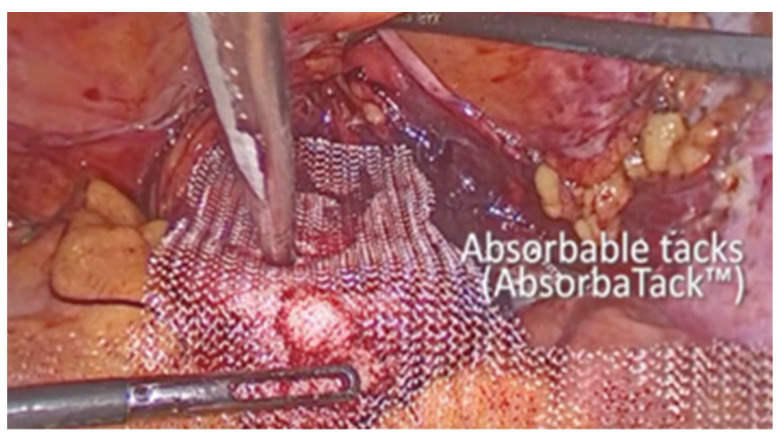
Fixation to the anterior vaginal wall.

**Figure 5:**
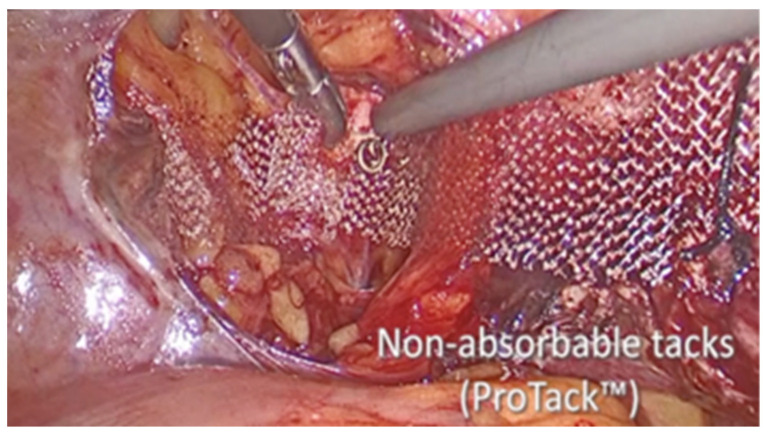
Fixation to the iliopectineal ligament.

**Figure 6:**
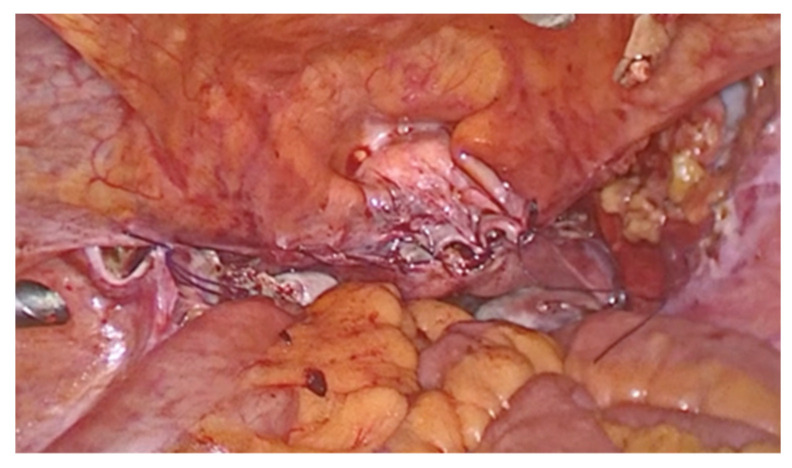
Final aspect.

The vesico-vaginal space was dissected as the procedure went on. With the aid of a retractor that was inserted into the vagina and held by an assistant, the anterior vaginal wall was exposed. An 8 × 15 cm polypropylene mesh, cut in a T shape, was introduced in the abdomen. First, many absorbable tacks were used to secure the short arm of the T to the anterior vaginal wall (AbsorbaTack fixation device; Medtronic, Dublin, Ireland) (Figure 4). Second, the ProTack fixation device (Figure 5 from Medtronic, Dublin, Ireland) was used to secure the lateral arms of the mesh to the iliopectineal ligaments with one or two nonabsorbable tacks on each side. Through the opposite trocar, the ProTack device was inserted to exert vertical pressure on the pectineal ligament and achieve the proper attachment of the tack. The procedure continued with the complete closure of the peritoneum using Vicryl 2.0 sutures (Ethicon) (Figure 6).

#### 2.1.2. Sacrospinous Ligament Fixation Technique Description

**Figure 7:**
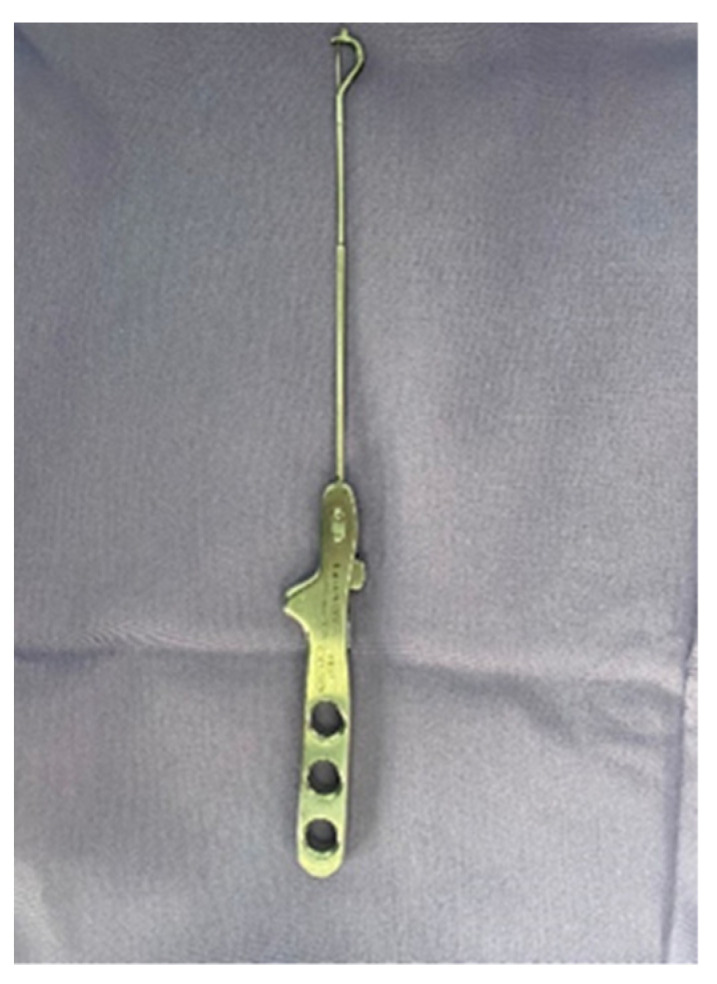
SSLF device.

The procedure was performed with the patient in the gynecological position under spinal anesthesia. A 16-French Foley catheter was inserted through the urethra in the bladder. During SSLF, after injecting the saline solution under the posterior vaginal wall, a longitudinal incision was made in the midline, 3 cm distal to the vaginal apex or cervix. One canal in the right pararectal space, created for to the surgeon’s finger, was formed under the vaginal wall, horizontally in the direction of the pelvic side wall and directly next to the right and left sacrospinous ligament. The ligament is a strong, triangular fibrous band that extends from the sacrum (the triangular bone at the base of the spine) to the ischial spine (a bony prominence in the pelvis). Two nonabsorbable, polypropylene monofilament 2.0 sutures were anchored to the right and left sacrospinous ligaments using the SSLF-specific device (Figure 7). The sutures were tied securely to anchor the vaginal apex to the sacrospinous ligament. The vaginal incision was closed with a Vicryl 2.0 suture.

### 2.2. Statistical Analysis

Data collection was performed using the Mediflux software 1.1 (developed by ORIGINi company, Amsterdam, The Netherlands). Statistical evaluations were conducted using Python 3.9.13 (Python Software Foundation). For the data manipulation the Pandas library was used, and the SciPy library was applied for statistical computations.

Descriptive statistics were performed to encapsulate the demographic and clinical features of the patient groups. Means and standard deviations (SD) were used to summarize continuous variables, while counts and percentages were used to represent categorical variables.

The Shapiro–Wilk test was employed to verify normality assumptions. To compare the means of the continuous and ordinal variables among the two patient groups, we applied the Mann–Whitney U test *p*-value.

With respect to categorical variables, Fisher’s exact test was primarily employed due to the small sample sizes of the groups. However, the Chi-square test was used in situations where the expected frequencies in each cell of the contingency table were adequate.

A *p*-value less than 0.05 was deemed statistically significant across all analyses.

## 3. Results

The demographic statistics and clinical characteristics of the 160 patients included in the study are presented in Table 1. The patients were divided into two groups based on the performed surgical intervention: 82 (51.25%) in the “SSLF” group and 78 (48.75%) in the “LP” group.

**Figure 8:**
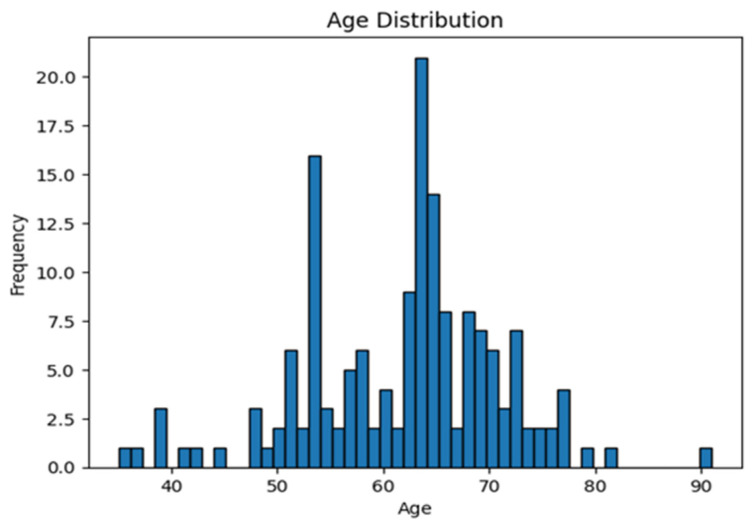
Group age histogram.

Significant differences were observed in age, BMI, and postmenopausal status between the two groups. The mean age of patients in the SSLF group was 64.56 years, higher than the mean age of 59.16 years in the LP group (*p* = 0.002) (Figure 8). The mean BMI for the SSLF group was 28.59, slightly higher than the LP group’s mean BMI of 27.60 (*p* = 0.003). Regarding menopausal status, there was a significantly higher proportion of postmenopausal women in the SSLF group (93.90%) compared to the LP group (74.35%) (*p* < 0.001).

In summary, patients undergoing SSLF surgery were significantly older, had slightly higher BMI and were more likely to be postmenopausal compared to those undergoing LP surgery.

With regard to the frequency of concomitant posterior colporrhaphy procedures, in the SSLF group 29.27% (24 out of 82) had undergone a concomitant posterior colporrhaphy, while in the LP group this rate was slightly lower at 24.36% (19 out of 78).

Table 2 summarizes the perioperative outcomes of the two surgical techniques. The Delta Hb, which represents the change in the hemoglobin value between pre- and post-operation levels, was found to be 1.01 (SD: 0.41) in the entire study group of 160 patients. In the subgroup analyses, the SSLF group had a Delta Hb of 1.04 (SD: 0.42), whereas the LP group (N2 = 78, 48.75% of the study group) recorded a Delta Hb of 0.98 (SD: 0.40), with no statistically significant difference between the two subgroups (*p* = 0.516).

Regarding the mean length of hospital stay, the entire study group averaged 2.34 days (SD: 0.47). The SSLF group had a slightly shorter mean hospital stay of 2.18 days (SD: 0.39) as compared to the LP group, which averaged 2.50 days (SD: 0.50), a difference found to be statistically significant (*p* < 0.001).

Lastly, the mean operating time was measured for both surgical techniques. The study group had a mean operating time of 47.00 min (SD: 18.35) overall. When divided by technique, the SSLF group had a significantly shorter operating time, averaging 32.20 min (SD: 6.28), compared to the LP group which took 62.56 min on average (SD: 13.23, *p* < 0.001).

In the evaluation of postoperative complications between the SSLF group and the LP group, differences were found to be statistically insignificant for most complications (Table 3). DN urgency was experienced by 6.87% of the overall study group, with a similar occurrence between the SSLF (N1 = 5, 6.10%) and LP group (N2 = 6, 7.69%), *p* = 0.93151.

Comparable trends were found for de novo cystocele (8.75% overall, 8.54% in SSLF vs. 8.97% in LP, *p* = 0.85561) and de novo rectocele (5.62% overall, 4.88% in SSLF vs. 6.41% in LP, *p* = 0.74162).

Pelvic pain was present in 5.00% of all patients, but was notably more frequent in the SSLF (7, 8.54%) than in the LP group (1, 1.28%), although the difference was not statistically significant (*p* = 0.06422). Similarly, constipation was experienced by 6.87% of patients, with a greater occurrence in the SSLF (9, 10.87%) compared to the LP group (2, 2.56%), *p* = 0.05732.

Dyspareunia occurred in 4.37% of all patients, slightly more frequently in the SSLF (6, 7.32%) than the LP group (1, 1.28%), but without significant difference (*p* = 0.11762). Lastly, de novo stress urinary incontinence (DN SUI) was seen in 8.75% of all patients, evenly distributed between the SSLF (8, 9.76%) and LP groups (6, 7.69%), *p* = 0.85561.

These results indicate that both surgical procedures have comparable postoperative complication rates, with no significant difference in the occurrence of any specific complication.

The results pertaining to the outcomes of the surgical procedures are presented in Table 4. The cure rate was high in both the SSLF group (N1 = 82, 51.25%) and the LP group (N2 = 78, 48.75%). Specifically, 95.12% of the patients (78 out of 82) in the SSLF group and 93.59% of the patients (73 out of 78) in the LP group were cured post-surgery, leading to an overall cure rate of 151 out of 160 patients. This difference in cure rates was not statistically significant (*p* = 0.6741, Chi-square test).

On the other hand, the failure rate was found to be low in both groups. Specifically, the failure rate was 4.88% (4 out of 82 patients) in the SSLF group and 6.41% (5 out of 78 patients) in the LP group, resulting in an overall failure rate of 9 out of 160 patients.

These results demonstrate that both surgical procedures have comparable outcomes, with high cure rates and low failure rates, reinforcing their effectiveness in treating pelvic organ prolapse.

In the conducted logistic regression analysis, none of the predictor variables—age, parity, BMI, postmenopausal status, or the type of surgical procedure performed—appeared to significantly influence the likelihood of treatment failure (*p* > 0.05 for all predictors) both in the SSLF group and LP group. Logistic regression analysis was also employed to assess the potential impact of concomitant surgery on surgery complications that could possibly be the result of the posterior colporrhaphy (pelvic pain, constipation and dyspareunia). The analysis revealed that the presence of concomitant surgery did not significantly influence the likelihood of these specific surgical complications in either of the groups (*p* = 0.635 for SSLF group; *p* = 0.976 for LP group).

Validated questionnaires such as the Prolapse Quality of Life (P-QOL) were completed before surgery and at follow-up visits. The responses are presented in Table 5. There are not significant differences between the answers of two groups.

The post-operatory UDI-6 questionnaire was offered for completion just for patients that presented de novo SUI or de novo urgency as postoperative complications. There was no significant variation between the mean scores of the two study groups (SSLF group: 55.97 vs. LP group: 53.88, *p* = 0.822).

## 4. Discussion

In our prospective study of 160 patients, the cure rate was high in both the SSLF group (N1 = 82, 51.25%) and the LP group (N2 = 78, 48.75%). Specifically, 95.12% of the patients (78 out of 82) in the SSLF group and 93.59% of the patients (73 out of 78) in the LP group were cured post-surgery, leading to an overall cure rate of 151 out of 160 patients, with no differences between the two groups in either anatomical and subjective success and surgical failure rates.

Noe et al., in association with 11 medical institutions, reported a total success rate for apical repair of 96.9%, with no mesh exposure or mesh-related complications at 12–18 months of follow-up (n = 264) [10,11]. Noe et al. compared 44 pectopexy patients with 41 sacropexy patients’ postoperative and intermediate-term follow-up outcomes. They reported that there was no difference between the apical descensus relapse and de novo rectocele rates. The lateral defect cystocele and de novo constipation rates were also reduced in the laparoscopic pectopexy group. Additionally, they observed that the anterior and lateral compartments were protected by the pectopexy [9]. Biler et al. [12] reported no recurrent prolapse or mesh exposure at a 6-month follow-up after laparoscopic pectopexy and Tahaoglu et al. found no evidence of recurrent prolapse after laparoscopic pectopexy at a 6-month follow-up in 22 patients [13].

Failure was determined by Favre-Inhofer et al. [14] as anatomical prolapse POP-Q > 1 or repeated surgery after analyzing the outcomes of 59 patients following SSLF at two follow-up intervals. They discovered no relapses during a 5- to 10-year follow-up period and showed an apical recurrence rate of 3% at 1–5 years after surgery. The most frequent site of recurrence was the anterior vaginal wall, with 9 (26%) and 4 (16%) occurrences at 1–5 years and 5–10 years of follow-up, respectively [14], and in our study the failure rate was higher, 4.88% (4 out of 82 patients) in the SSLF group and 6.41% (5 out of 78 patients) in the LP group.

The apical relapse rates in the study by Astepe et al. (14% in the SSLF group vs. 11.1% in the LP group) were comparable in both groups [15]. The SSLF group had greater de novo cystocele rates of 25.6% vs. 8.3% in the LP group. This could be explained by the fact that the SSLF group’s vaginal axis deviated to the right and posterior, and the body’s center of gravity shifted to the anterior, placing more weight on the anterior compartment with no appreciable differences in the numbers of de novo rectocele between the groups [15]. In our study the rates for de novo cystocele were similar for both surgeries: 8,54% for SSLF group and 8,97% in the LP group.

The vaginal axis may be closer to the original anatomic position if SSLF is performed bilaterally. The patients’ sexual life can be effectively improved by this. Mehmet Baki et al. [16] demonstrated that vaginal bilateral SSLF with mesh established adequate pelvic support for genital organ prolapse up to 18 months. Recent research, including the OPTIMAL [17] and OPUS [18] studies, indicated that anterior compartment refection with colposuspension in a stage 3 or 4 prolapse resulted in a better anatomic outcome in the anterior compartment. Their anterior compartment recurrence rate was 25%, and 19%, respectively [17,18].

According to Weemhoff et al. [19], when SSLF and anterior colporrhaphy are combined, the chance of recurrence is higher. Their initial theory was that the weaker compartment would experience a recurrence caused by a different intra-abdominal pressure distribution. The second theory was that because the prolapse is more prominent in one compartment than another, the first clinical examination was undervalued there. The other compartment is exposed when the one that is primarily implicated is treated [19].

Due to the mesh position in LP and suture placement in SSLF, the risk of de novo constipation is negligible compared to SCP (laparoscopic sacrocolpopexy) or SCerP (sacrocervicopexy) [10,15,20]. In the current study, we found a high rate of constipation in the SSLF group, 9 patients out of 82. After SSLF, the anal functions can be impacted. The anal sphincter’s functions may be negatively impacted by a pudendal nerve injury. 14 out of 200 individuals in one study experienced de novo anal incontinence [21].

Injury to the pudendal nerve and its branches, which surround the sacral plexus, can explain buttock pain. Following SSLF, a 10% rate was recorded by Ping Wang et al. [22], but this number dropped to 0 at 6 and 12 months postoperatively. One incidence of persistent thigh pain was reported by Feiner et al. and which gradually settled after 12 months [14]. A similar rate was reported by Szymczak et al.’s [8] study within 30 days of discharge from the hospital. At follow-up visits, three (5%) patients reported persistent buttock pain [11].

In the current study, we found a higher de novo SUI rate in the SSLF group (9.76%). The insertion of a continence procedure to a prolapse repair surgery could reduce the incidence of postoperative urinary incontinence but this advantage needs to be balanced against possible differences in costs and side effects [23].

In the LP group, Fei Chi Chuang found a greater incidence of postoperative SUI (n = 6, 33.3%). A preoperative urodynamic examination revealed that four of them had occult SUI. In cases when POP surgery is performed without a concurrent anti-incontinence therapy, occult SUI is a known risk factor for postoperative SUI [24]. Noe et al. identified low de novo SUI rates of 4.8% in the laparoscopic pectopexy group [5].

Pirtea et al. [25] presented a case of pelvic organ prolapse associated with stress urinary incontinence treated via laparoscopic pectopexy followed by Burch colposuspension with no intraoperative or postoperative complications and the follow-up assessment revealed an effective correction of the prolapse and of the SUI.

There was no difference between the postoperative de novo urge and stress urinary incontinence rates in the SSLF and LP groups in the Astepe et al. study [15].

Regarding intraoperative complications, Noe et al. [5] found five patients with severe complications—one hemorrhage and four cases of organ damage (three bladder lesions and one ureter injury). Biler et al. [12] reported one case of hemorrhage during LP (n = 16), which did not require blood transfusion and in one study one patient was reported with an intraoperative hemorrhage from corona mortis stopped by bipolar cautery [26].

An increased risk of genital prolapse is connected to obesity. Laparoscopic procedures are beneficial for patients who are obese in terms of postoperative morbidity and wound healing, but they can also be limited by the surgical field difficulties in balancing sufficient abdominal pressure and proper ventilation [5,27]. In contrast to sacrocolpopexy, pectopexy limits the surgical fields in the anterior pelvic space and is less influenced by obesity [24].

Despite the intuitive possibility of age, parity, and BMI affecting the treatment failure rates, the statistical analysis does not confirm this assumption. It is possible that other factors not included in this model may play a crucial role in predicting treatment failure, or that a larger dataset might be required to detect the potential subtle effects of these variables. Further research and data analysis are required to identify the key determinants of treatment failure rates.

Pelvic ligaments are used in a variety of surgical procedures to treat rectal and vaginal prolapse as well as urinary incontinence. The iliopectineal ligament is a strong structure for the fixation of sutures and meshes in place, respectively. For Burch colposuspension and lateral repair, the medial portions of this ligament have been used successfully as a support. The iliopectineal ligament is statistically substantially stronger than the sacrospinous ligament and the arcus tendinous of the pelvic fascia [5] according to Cosson et al. [28,29]. No similar study measuring the strength of ligaments on humans or animals has previously been published [8,30].

The laparoscopic pectopexy procedure is effective for making long-lasting repairs. The surgeon should be skilled in intra-corporal laparoscopic suture and knotting techniques as well as the anatomical characteristics of the female pelvis.

Limitations of the study include the fact that all procedures where performed by the same surgical team in a single center, which may be subject to bias. Other study limitations are represented by the small follow-up period and the small study population.

Given the logistical and financial constraints, particularly around the intricate organization and the costs of rigorous data management, using a prospective cohort study emerged as a more feasible and cost-effective approach than an RCT for this particular research.

## 5. Conclusions

In this prospective study with a mean of two years’ follow-up, the SSLF procedure and LP procedure were effective with a high cure rate and safety in the treatment of POP with a low rate of complications after 2 years of follow-up.

Pectopexy is a promising prolapse correction technique, although more research is still needed to determine its long-term outcomes. The vaginal sacrospinous ligament fixation maintains its value in prolapse surgery with the increasing importance of native tissue repair. Both SSLF and LP are effective in the treatment of POP, with favorable anatomical and subjective results and low rates of serious postoperative complications.

## Figures and Tables

**Table 1:** Clinical characteristics of the patients.

Parameter	Mean (95%CI) or N (%)			
	Study Group N = 160	SSLF Group N1 = 82 (51.25%)	LP Group N2 = 78 (48.75%)	*p-*Value
Age	61.93 (60.49–63.37)	64.56 (62.75–66.37)	59.16 (57.06–61.27)	0.002 ^1,^*
BMI	28.11 (27.73–28.49)	28.59 (27.73–28.49)	27.60 (26.89–28.32)	0.003 ^1,^*
Parity	2.64 (2.5–2.77)	2.63 (2.5–2.77)	2.64 (2.44–2.84)	0.097 ^1^
Post Menopause	135 (84.37%)	77 (93.90%)	58 (74.35%)	<0.001 ^1,^*
Pelvic Organ Prolapse Quantification (POP-Q) stages				
II	3 (1.86%)	0 (0.00%)	3 (3.84%)	*-*
III	117 (73.13%)	62 (75.60%)	55 (70.51%)	0.467 ^2^
IV	40 (25.01%)	20 (24.40%)	20 (25.64%)	0.855 ^2^

^1^ Mann–Whitney U test *p*-value; ^2^ Chi-square test; *p*-value; * statistically significant.

**Table 2:** Perioperative outcomes of the surgical techniques.

Outcome	N (SD)			
	Study Group N = 160	SSLF N1 = 82 (51.25%)	LP N2 = 78 (48.75%)	*p*-Value
Delta Hb	1.01 (0.41)	1.04 (0.42)	0.98 (0.40)	0.516 ^1^
Mean Hospital Stay	2.34 (0.47)	2.18 (0.39)	2.50 (0.50)	<0.001 ^1,^*
Operating Time	47.00 (18.35)	32.20 (6.28)	62.56 (13.23)	<0.001 ^1,^*

^1^ Mann–Whitney U test *p*-value; * statistically significant.

**Table 3:** Complications of surgical procedures.

Complications Postoperative	N (%)			
	Study Group N = 160	SSLF N1 = 82 (51.25%)	LP N2 = 78 (48.75%)	*p*-Value
DN Urgency	11(6.87%)	5(6.10%)	6(7.69%)	0.9315 ^1^
DN Cystocele	14(8.75%)	7(8.54%)	7(8.97%)	0.8556 ^1^
DN Rectocele	9(5.62%)	4(4.88%)	5(6.41%)	0.7416 ^2^
Pelvic Pain	8(5.00%)	7(8.54%)	1(1.28%)	0.0642 ^2^
DN Constipation	11(6.87%)	9(10.87%)	2(2.56%)	0.0573 ^2^
Dyspareunia	7(4.37%)	6(7.32%)	1(1.28%)	0.1176 ^2^
DN SUI	14(8.75%)	8(9.76%)	6(7.69%)	0.8556 ^1^

^1^ Chi-square test *p*-value; ^2^ Fisher’s exact test *p*-value.

**Table 4:** Outcomes of surgical procedures.

Outcome	N (%)			
	Study Group N = 160	SSLF N1 = 82 (51.25%)	LP N2 = 78 (48.75%)	*p*-Value
Cure	151 (94.37%)	78 (95.12%)	73 (93.59%)	0.674 ^1^
Failure	9 (5.63%)	4 (4.88%)	5 (6.41%)	
Relapse before two years	18 (11.25%)	10 (12.19%)	8 (10.25%)	0.150 ^2^

^1^ Fischer exact-test *p*-value; ^2^ Chi-square test.

**Table 5:** Pre-operatory and post-operatory P-QOL response in the study group.

	N (%)			
	Study Group N = 160	SSLF N1 = 82 (51.25%)	LP N2 = 78 (48.75%)	*p*-Value
Pre-operatory P-QOL response				
slightly	58 (36.25%)	28 (34.15%)	30 (38.46%)	0.623 ^1^
moderately	75 (46.87%)	39 (47.65%)	36 (46.15%)	0.875 ^1^
a lot	27 (16.88%)	15 (18.29%)	12 (15.38%)	0.676 ^1^
Post-operatory P-QOL response				
not at all	106 (66.25%)	55 (67.07%)	51 (65.38%)	0.868 ^1^
slightly	25 (15.62%)	15 (18.29%)	10 (12.82%)	0.388 ^1^
moderately	20 (12.50%)	8 (9.76%)	12 (15.38%)	0.342 ^1^
a lot	9 (5.62%)	4 (4.88%)	5 (6.41%)	0.741 ^1^

^1^ Fisher’s exact-test *p*-value.

## Data Availability

Data is contained within the article.
